# Preoperative concurrent chemoradiotherapy with cisplatin and docetaxel in patients with locally advanced non-small-cell lung cancer

**DOI:** 10.1038/sj.bjc.6601624

**Published:** 2004-03-02

**Authors:** H Katayama, H Ueoka, K Kiura, M Tabata, T Kozuki, M Tanimoto, T Fujiwara, N Tanaka, H Date, M Aoe, N Shimizu, M Takemoto, Y Hiraki

**Affiliations:** 1 Department of Internal Medicine II, Okayama University Hospital, 2-5-1 Shikata-cho, Okayama 700-8558, Japan; 2 Department of Surgery I, Okayama University Hospital, 2-5-1 Shikata-cho, Okayama 700-8558, Japan; 3 Department of Surgery II, Okayama University Hospital, 2-5-1 Shikata-cho, Okayama 700-8558, Japan; 4 Department of Radiology, Okayama University Hospital, 2-5-1 Shikata-cho, Okayama 700-8558, Japan

**Keywords:** induction chemoradiotherapy, cisplatin, docetaxel, non-small-cell lung cancer

## Abstract

The objective of this study was to assess the feasibility and effectiveness of an induction chemoradiotherapy regimen followed by surgery in patients with locally advanced non-small-cell lung cancer (LA-NSCLC). A total of 22 patients with LA-NSCLC were treated with induction chemoradiotherapy consisting of cisplatin (40 mg m^−2^) and docetaxel (40 mg m^−2^) given on days 1, 8, 29 and 36 plus concurrent thoracic irradiation at a dose of 40–60 Gy (2 Gy fraction^−1^ day^−1^). Surgical resection was performed within 6 weeks after completion of induction therapy. Objective response to the induction therapy was obtained in 16 patients (73%). In all, 20 patients (91%) underwent surgery and complete resection was achieved in 19 patients (86%). Pathological downstaging and pathological complete response were obtained in 14 (64%) and five (23%) patients, respectively. With a median follow-up period of 32 months, the calculated 3-year overall and progression-free survival rates were 66 and 61%, respectively. It is noteworthy that the 3-year overall survival rate in 14 patients achieving pathological downstaging was extremely high (93%). Toxicity was manageable with standard approaches. No treatment-related deaths occurred. This combined modality treatment is feasible and highly effective in patients with LA-NSCLC. The results warrant further large-scale study to confirm the effectiveness of this regimen.

Since the majority of patients with locally advanced non-small-cell lung cancer (LA-NSCLC) develop distant metastases, the treatment outcomes of patients receiving surgery or radiotherapy alone are extremely poor. In previous reports, 5-year survival rates of LA-NSCLC patients undergoing locoregional treatment alone ranged from 5 to 15% (Pearson *et al*, 1982; Martini and Flehinger, 1987; Mountain, 1988; Shields, 1990; Cox *et al*, 1991). Therefore, a combined modality approach to control both local tumour and distant micrometastasis is required to improve the treatment outcome. A variety of multimodality therapies that include chemotherapy, surgery and/or radiotherapy have recently been assessed in clinical trials. Use of postoperative chemotherapy or radiotherapy has not shown an apparent survival benefit to date (The Lung Cancer Study Group, 1981; Pisters *et al*, 1994; Keller *et al*, 2000). Although Le Chevalier *et al* (2003) recently reported a statistically significant prolongation of survival in a large-scale randomised study of postoperative chemotherapy, the advantage was extremely limited. On the other hand, preoperative chemotherapy resulted in a definite survival advantage in a few randomised trials comparing preoperative chemotherapy plus surgery with surgery alone in LA-NSCLC patients (Rosell *et al*, 1994; Roth *et al*, 1994). Local recurrence rates in these trials were considerably high, however. For example, Rosell *et al* (1994) reported a local recurrence rate of 54% in patients who received preoperative mitomycin, ifosfamide and cisplatin. Similarly, in our previous study of preoperative cisplatin and irinotecan chemotherapy, the local recurrence rate was 33% overall and 50% in patients being treated for disease relapse (Date *et al*, 2002). Then, we considered that further prolongation of survival might be obtained by improvement of local control, adding concurrent thoracic irradiation to induction chemotherapy. In several recent reports, preoperative chemoradiotherapy in patients with LA-NSCLC was shown to be feasible and effective, although the new drugs developed in the 1990s such as irinotecan and docetaxel were not included (Albain *et al*, 1995; Choi *et al*, 1997; Eberhardt *et al*, 1998; Thomas *et al*, 1999). We have already confirmed the feasibility and effectiveness of concurrent chemoradiotherapy using cisplatin and docetaxel in patients with unresectable LA-NSCLC (Kiura *et al*, 2003), whereas a combination of cisplatin, irinotecan and concurrent thoracic irradiation has been reported to be toxic and unacceptable (Yokoyama *et al*, 1996). Based on these results, the present study was planned to assess the feasibility and effectiveness of this chemoradiotherapy as preoperative treatment in patients with LA-NSCLC.

## PATIENTS AND METHODS

### Patient selection

Previously untreated patients with histologically confirmed stage IIIA or IIIB NSCLC, with measurable disease, were eligible for the study. In this study, a mediastinal lymph node ⩾10 mm along the short axis by CT scan was defined as a metastatic lymph node. The other inclusion criteria were age ⩽75 years, Eastern Cooperative Oncology Group (ECOG) performance status (PS) of 0–2 (Oken *et al*, 1982), and adequate functional reserves of bone marrow (leucocyte count >4000 *μ*l^−1^, platelet count >100 000 *μ*l^−1^), liver (serum bilirubin level <1.5 mg dl^−1^, aspartate aminotransferase and alanine aminotransferase levels (AST/ALT) <2 times the upper normal limit), kidney (serum creatinine level <1.5 mg dl^−1^, creatinine clearance >60 ml min^−1^) and lung (arterial oxygen pressure (PaO_2_) >60 Torr). Patients with concomitant malignancies, supraclavicular lymph node involvement, or malignant pleural or pericardial effusion were excluded from the study. Written informed consent was obtained from all patients.

### Evaluations

Staging procedures included medical history and physical examination, complete blood cell counts (CBC), standard blood chemistry profile, 24-h urine creatinine clearance (Ccr), electrocardiogram, chest radiography, computed tomography (CT) scans of the chest and upper abdomen, magnetic resonance imaging (MRI) of the brain, fibre optic bronchoscopy and radionuclide bone scan. Magnetic resonance imaging of the chest was required if mediastinal invasion was suspected.

During treatment, CBC was repeated two to three times a week, and blood chemistry tests, Ccr evaluations and chest radiograph were repeated at least once a week. Chest CT scans were repeated after each chemotherapy course. After completion of combined modality treatment, each patient was restaged with all tests used for the initial work-up, and followed monthly with chest radiographs. CT scans were repeated every 3 months.

### Treatment plan

Chemotherapy was administered on days 1, 8, 29 and 36. Patients were premedicated with dexamethasone (8 mg) and granisetron (3 mg) or ondansetron (4 mg) immediately prior to cisplatin administration. Cisplatin 40 mg m^−2^ diluted in 300 ml of physiological saline was given as a 1-h i.v. infusion, followed by docetaxel 40 mg m^−2^ dissolved in 500 ml of physiological saline as a 1-h i.v. infusion. Patients then received hydration with 2000 ml of physiological saline. If leucocyte count was less than 3000 *μ*l^−1^ or platelet count less than 100 000 *μ*l^−1^ on day 29, chemotherapy was postponed until recovery. No dose attenuations were planned for reductions in leucocyte or platelet counts on days 8 or 36. Radiotherapy was started on the first day of chemotherapy using a linear accelerator (6–10 MeV). A total radiation dose of 40 Gy was planned with conventional fractionation (2 Gy day^−1^). Dose escalation of radiotherapy was allowed for poorly responding tumours. The original volume included the site of primary tumour with a margin of 2 cm around the mass and the ipsilateral hilum, and the whole width of the mediastinum with a margin of 1 cm around the radiographically visible region of involvement extending inferiorly to 3 cm below the carina or 2 cm below the radiographically demonstrated tumour mass. The original volume was treated with an anterior–posterior parallel-opposed pair of portals at doses of 40 or 46 Gy. For poor responding patients, an additional 20 Gy dose was administered to the boost volume, including the sites of primary tumour and involved lymph nodes. The boost volume was treated with a pair of oblique fields to keep cumulative radiation dose to the spinal cord at less than 46 Gy.

Following induction chemoradiotherapy, patients were evaluated for response based on a chest radiograph and CT scans. Patients without progressive disease were to have surgery within 6 weeks of completing induction therapy. The surgical procedure was determined on the basis of disease extent before induction treatment. Lobectomy was preferred; however, bilobectomy, sleeve resection, or pneumonectomy was performed in cases requiring those procedures because of primary tumour invasion. Resection with reconstruction of the chest wall was performed if necessary. The bronchial stump was covered with intercostal muscle pedicle.

Postoperative treatment was left to the physician's discretion. Usually, if apparent residual tumour was left or viable cells were found in the surgically resected specimens, further chemotherapy was given.

### Response, survival and toxicity assessments

Response was assessed using ECOG criteria (Oken *et al*, 1982), with some modification, as follows: complete response (CR), disappearance of all tumour at the end of induction therapy; partial response (PR), a ⩾50% reduction in the sum of the products of two perpendicular dimensions of all measurable lesions; stable disease (SD), a <50% reduction and <25% increase in the sum of the products of two perpendicular dimensions of all measurable lesions; progressive disease (PD), a ⩾25% enlargement of tumour lesion or the appearance of any new lesions. Survival was assessed on an ‘intent-to-treat’ basis, with survival time defined as the period from initiation of chemoradiotherapy to death or the last follow-up evaluation. The survival curve was calculated by the Kaplan and Meier (1958) method. Toxicity was assessed by the National Cancer Institute-Common Toxicity Criteria (NCI-CTC) (NCI-CTC version 2.0, 1998). Statistical analyses were performed using an SPSS Base System and Advanced Statistics Program (SPSS Inc., Chicago, IL, USA).

## RESULTS

### Patient characteristics

Between August 1998 and August 2001, 22 patients with stage IIIA or stage IIIB NSCLC were treated with this combined modality treatment. The baseline patient characteristics are listed in Table 1

**Table 1:** Patient characteristics

	**No. of patients**	
No. of patients evaluated	22	
Median age in years (range)	60	(30–73)

*Sex*		
Male	15	
Female	7	

*ECOG PS*		
0	11	
1	11	

*Histology*		
Adenocarcinoma	11	
Squamous cell carcinoma	11	

*Stage of disease*		
IIIA	14	
T1N2M0		3
T2N2M0		7
T3N2M0		4
IIIB	8	
T4N0M0		2
T4N1M0		1
T4N2M0		4
T3N3M0		1

ECOG=Eastern Corporative Oncology Group; PS=performance status.

. Median age was 60 years and PS was 0 or 1 in all patients. In all, 18 patients had N2 disease, of whom nine had histologically proven N2 disease by mediastinoscopy or transbronchial needle aspiration biopsy. One patient had N3 disease in the contralateral mediastinal lymph node. Seven patients had T4 disease consisting of aortic invasion in three patients, pulmonary artery invasion in three, and mediastinal invasion in one, which were confirmed by enhanced chest MRI.

### Induction therapy: response and toxicities

Out of 22 evaluable patients, 11 completed the planned chemoradiotherapy treatment. Chemotherapy dose was modified due to toxicity in 11 patients. Docetaxel dose was reduced on days 29 and 36 in four patients. Four patients did not receive chemotherapy on day 36 due to neutropenia in three patients and diarrhoea in one. In three patients, chemotherapy was omitted on both days 29 and 36 and only radiation therapy was accomplished, because of anaphylactic reaction to docetaxel, paralytic ileus and patient refusal in one patient each. The total radiation dose was 40 Gy in 10 patients, 46 Gy in nine and 60 Gy in three. Clinical response to induction therapy was PR in 16 patients (73%) and SD in six (27%). Toxicities experienced during induction therapy are listed in Table 2

**Table 2:** Toxicity of induction therapy (*n*=22)

	**Grade**				
	**1**	**2**	**3**	**4**	**% of toxicities ⩾grade 3**
Leukopenia	2	6	10	3	59.1
Neutropenia	2	5	9	4	59.1
Anaemia	10	7	1	0	4.5
Thrombocytopenia	9	0	0	0	0
Nausea/vomiting	13	3	2	0	9.1
Diarrhoea	1	3	1	0	4.5
Constipation	0	0	0	1	4.5
Hepatic	8	2	0	0	0
Renal	3	0	0	0	0
Cardiac	0	0	0	1	4.5
Pulmonary	0	0	0	0	0
Oesophagitis	5	5	1	0	4.5
Allergy	0	0	0	1	4.5

Toxicity was assessed and graded using NCI common toxicity criteria (National Cancer Institute Common Toxicity Criteria, version 2.0, 1998).

. Grades 3 and 4 leukopenia and neutropenia were each observed in 13 patients; however, no febrile neutropenia occurred. Nonhaematological toxicities were primarily gastrointestinal effects, although three patients had serious effects including congestive heart failure with atrial fibrillation, paralytic ileus and anaphylactic reaction to docetaxel in one patient each.

### Surgery and pathologic response

Two of 22 patients did not undergo surgical resection because of congestive heart failure (*n*=1) and patient refusal (*n*=1). For 20 patients who had surgery, the median time from the end of induction therapy to surgery was 37 days (range, 25–59 days). Surgical procedures included lobectomy in 16 patients, sleeve lobectomy in two and bilobectomy and pneumonectomy in one each. One patient who had contralateral mediastinal lymph node metastasis before beginning chemoradiotherapy underwent contralateral mediastinal lymph node resection (R3). In all, 19 patients had complete tumour resection with microscopically negative margins. In one patient, residual tumour remained microscopically at the resected margin after surgery. Pathological downstaging was obtained in 14 patients (64%) and pathological CR (no viable tumour cells in surgical specimens) was achieved in five of those 14 patients (23%), the latter five of whom were determined to have obtained PR by CT scan after induction therapy.

### Postoperative treatment

Among 20 patients undergoing surgical resection, 12 received no postoperative chemotherapy or radiotherapy until disease progression. Six patients received postoperative chemotherapy consisting of cisplatin and docetaxel, of whom four had two treatment courses and two had one course because of toxicity (haemothorax and diarrhoea in one patient each). One patient whose tumour was not downstaged underwent further chemoradiotherapy comprising two courses of cisplatin and docetaxel with concurrent thoracic irradiation at a dose of 20 Gy. Another patient, whose tumour could not be completely resected, underwent bronchial artery infusion of cisplatin, mitomycin C and vindesine with concurrent thoracic irradiation at a dose of 20 Gy. Thus, two patients of 22 received a total radiation dose of 60 Gy.

### Postoperative complications

The major postoperative complication was pulmonary toxicity. One patient receiving 60 Gy radiotherapy developed haemothorax during postoperative chemotherapy. He was successfully managed by thoracic tube drainage and was alive without recurrence at the time of this report. Two patients had massive pleural effusions and were successfully treated by tube drainage and pleural adhesion therapy. Six patients experienced radiation pneumonitis (grade 2 in four patients and grade 3 in two) and were treated with prednisolone. These toxicities were reversible.

### Survival and pattern of relapse

**Figure 1:**
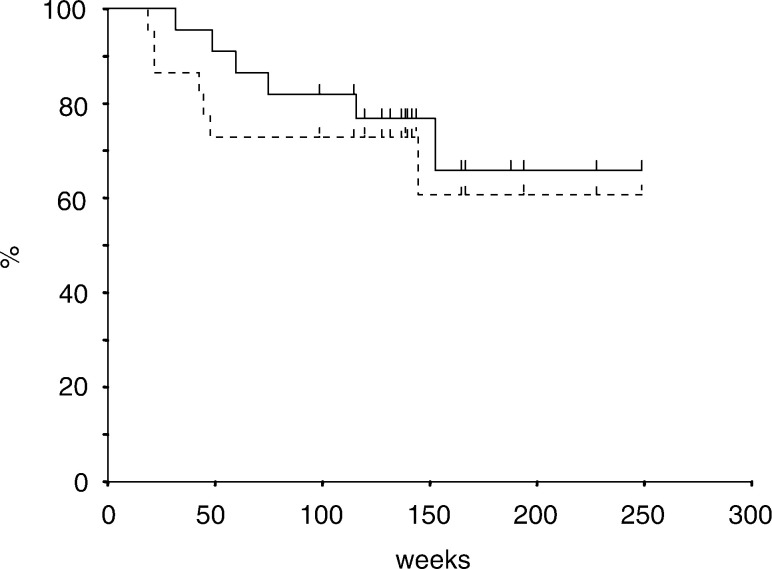
Overall and disease-free survival curves for 22 enrolled patients. Estimated 3-year survival and disease-free survival rates were 66 and 61%, respectively.

**Figure 2:**
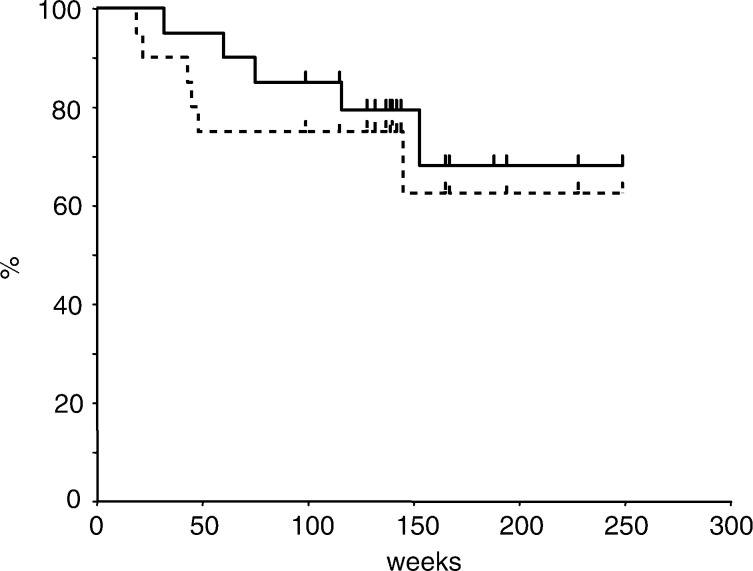
Overall and disease-free survival curves for 20 patients who underwent surgical resection. Estimated 3-year survival and disease-free survival rates were 68 and 63%, respectively.

**Figure 3:**
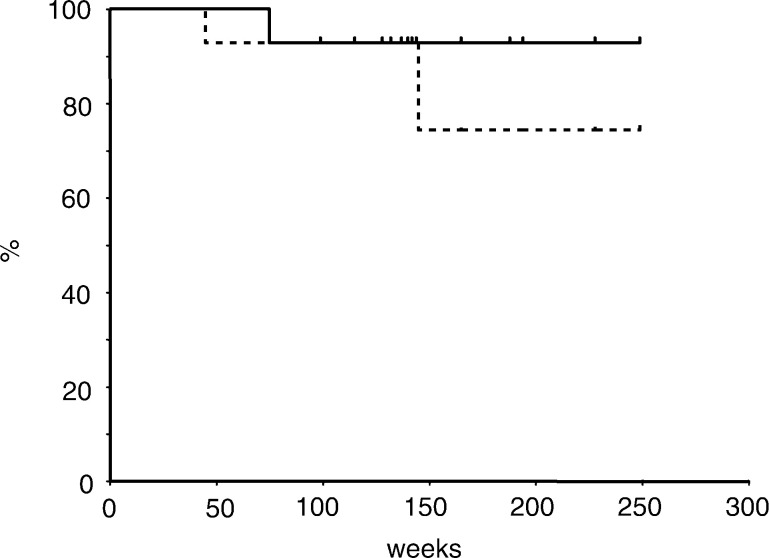
Overall and disease-free survival curves for 14 patients who achieved pathological downstaging of disease. Estimated 3-year survival and disease-free survival rates were 93 and 74%, respectively.

At a median follow-up time of 32 months, seven of 22 patients have had disease relapse. Of those seven patients, six died of disease progression and one developed solitary brain metastasis and is still alive. Progression of local tumour was observed in only two of the six patients who died of cancer. The initial failure sites in the other four patients were supraclavicular lymph node, para-aortic lymph node, lung and lumbar spine in one patient each. A total of 15 patients are currently alive with no evidence of recurrent disease. The 3-year overall and progression-free survival rates were 66 and 61% in 22 enrolled patients (Figure 1

), 68 and 63% in 20 patients who had surgical resection (Figure 2

) and 93 and 74% in 14 patients who achieved downstaging of disease (Figure 3

), respectively. Five patients achieving pathological CR are currently alive, although one has developed a solitary brain metastasis.

## DISCUSSION

The objective of this study was to investigate the feasibility and effectiveness of an induction chemoradiotherapy regimen consisting of cisplatin, docetaxel and concurrent thoracic radiation followed by surgery in patients with LA-NSCLC. Results confirmed that this combined modality treatment was well tolerated. In other recent reports of induction chemoradiotherapy followed by surgery, the frequencies of treatment-related deaths were approximately 2% during induction therapy and between 4 and 7.5% after surgery (Albain *et al*, 1995; Choi *et al*, 1997; Eberhardt *et al*, 1998; Thomas *et al*, 1999). Therefore, it is notable that none of the patients in the study reported herein experienced treatment-related deaths during induction therapy or after surgery. In the present study, the major toxicities of induction therapy, which included leukopenia, congestive heart failure, paralytic ileus and anaphylactic reaction to docetaxel, were manageable with standard approaches. The main postoperative toxicities were pulmonary complications such as haemothorax, massive pleural effusion and pneumonitis, which were also successfully managed by standard treatment.

Efficacy results after induction chemoradiotherapy were good, with 73% of patients responding and a 3-year survival rate of 66%. These findings are superior to those achieved in other studies in patients with LA-NSCLC. For example, 3-year survival rates of 27% for stage IIIA and 24% for stage IIIB patients were reported by Albain *et al*, 37% by Choi *et al* and 35% for stage IIIA and 26% for stage IIIB patients by Thomas *et al* (Albain *et al*, 1995; Choi *et al*, 1997; Thomas *et al*, 1999).

To improve local control, we adopted concurrent chemoradiotherapy as induction therapy. In the 1990s, a few randomised trials reported a survival advantage with induction chemotherapy followed by surgery, compared to surgery alone in patients with LA-NSCLC (Rosell *et al*, 1994; Roth *et al*, 1994). We also previously reported that induction chemotherapy with cisplatin and irinotecan was effective in 15 patients with LA-NSCLC, having pathologically confirmed mediastinal metastases (Date *et al*, 2002). The 3-year disease-free and overall survival rates were 24 and 40%, respectively, which were considered encouraging. However, pathological downstaging was obtained in only two patients (13%) and pathological CR in one (7%). Furthermore, initial disease relapse occurred at the local site in half of the patients who had disease relapse (Date *et al*, 2002). These results suggested that methods to achieve better local control were needed to improve the overall outcome for patients with LA-NSCLC.

Based on these findings, the study reported herein used induction chemoradiotherapy followed by surgery in attempts to improve the local tumour control. This approach resulted in downstaging of disease in 59% of the patients, which is similar to the result (67%) reported by Choi *et al* (Choi *et al*, 1997; Date *et al*, 2002), and significantly better than that in our previous study (13%, *P*=0.007). The pathological CR rate of 23% in the present study seemed to be improved in comparison with results of our previous study (7%, *P*=0.40) and Choi *et al* (10%), and comparable to those of Eberhardt *et al* (26%) and Thomas *et al* (18%) (Choi *et al*, 1997; Eberhardt *et al*, 1998; Thomas *et al*, 1999; Date *et al*, 2002). Moreover, local recurrence developed in only two (29%) of seven relapsed patients. Overall, these results indicate that considerably good local control can be achieved by using concurrent chemoradiotherapy as induction.

Several other factors may have contributed to the positive findings in this study. First, in an attempt to treat distant micrometastases, we used a two-drug combination of cisplatin plus docetaxel– one of the most active regimens for advanced NSCLC (Schiller *et al*, 2002). Cisplatin, the key drug in the treatment of NSCLC (Le Chevalier *et al*, 1999), has potent radiosensitising effects (DeWit, 1987), and docetaxel, a new active drug for NSCLC (Creane *et al*, 1999), also is a radiosensitising agent (Caffo, 2001). We thus considered that cisplatin plus docetaxel would be an excellent regimen to incorporate in concurrent chemoradiotherapy for LA-NSCLC. We previously reported that combined modality therapy using cisplatin and docetaxel with concurrent thoracic irradiation was feasible and effective in patients with unresectable LA-NSCLC (Kiura *et al*, 2003). The regimen achieved good control of local tumour and also of distant metastases. In previous studies of induction chemoradiotherapy, cisplatin-based regimens that included older agents such as etoposide, vinblastine, or vindesine were frequently used as induction chemotherapy (Albain *et al*, 1995; Choi *et al*, 1997; Eberhardt *et al*, 1998; Thomas *et al*, 1999). However, recent randomised trials showed cisplatin-based combinations that incorporated newer drugs such as docetaxel, paclitaxel, and vinorelbine, which were more effective than older cisplatin-based regimens in patients with advanced NSCLC (Le Chevalier *et al*, 1994; Bonomi *et al*, 2000; Kubota *et al*, 2002). These findings thus support the use of a cisplatin plus docetaxel combination in chemoradiotherapy for LA-NSCLC.

A second factor to consider, regarding the positive results in this study, is that the administration of both cisplatin and docetaxel was fractionated, that is, given on days 1, 8, 29 and 36. This schedule increased the opportunities for simultaneous administration of chemotherapy and radiotherapy, and may thus have increased the radiosensitising effects of chemotherapy. Furthermore, fractionated drug administration may increase chemotherapy dose intensity. In the present study, the projected dose intensity of both cisplatin and docetaxel was 20 mg m^−2^ week^−1^. In actuality, 18 mg m^−2^ week^−1^ of cisplatin and 17 mg m^−2^ week^−1^ of docetaxel was administered despite th use of concurrent thoracic irradiation. These doses are almost equal to those used for patients with advanced NSCLC, who are not receiving thoracic irradiation.

Third, although hyperfractionated radiotherapy has been the preferred approach in recent trials of preoperative chemoradiotherapy (Choi *et al*, 1997; Eberhardt *et al*, 1998; Thomas *et al*, 1999), results from a recent large-scale randomised trial in LA-NSCLC showed no superiority of hyperfractionation over standard fractionation (Curran *et al*, 2003). Thus, we used standard fractionation in the study reported herein, which allowed safe delivery of sufficient radiation doses, while avoiding severe radiation-induced oesophagitis or pneumonitis.

In conclusion, combined modality treatment consisting of induction therapy with cisplatin, docetaxel and concurrent thoracic irradiation followed by surgery is feasible and effective in patients with LA-NSCLC. A large-scale randomised trial is warranted to confirm these promising results.
